# Transcorneal delivery of topically applied silver nanoparticles does not delay epithelial wound healing

**DOI:** 10.1016/j.impact.2021.100352

**Published:** 2021-08-24

**Authors:** Soohyun Kim, Brooke L. Gates, Maggie Chang, Kent E. Pinkerton, Laura Van Winkle, Christopher J. Murphy, Brian C. Leonard, Philip Demokritou, Sara M. Thomasy

**Affiliations:** a Department of Surgical and Radiological Sciences, School of Veterinary Medicine, University of California - Davis, Davis, CA 95616, USA; b Center for Health and the Environment, University of California, Davis, CA 95616, USA; c Department of Anatomy, Physiology and Cell Biology, School of Veterinary Medicine, University of California, Davis, CA 95616, USA; d Department of Ophthalmology and Vision Science, School of Medicine, University of California, Davis, CA 95616, USA; e Center for Nanotechnology and Nanotoxicology, HSPH-NIEHS Nanosafety Center, Department of Environmental Health, Harvard T.H. Chan School of Public Health, Harvard University, 665 Huntington, Boston, MA 02115, USA

**Keywords:** Silver nanoparticles, Silver silica nanoparticles, Corneal epithelial wound healing, Keratocyte-fibroblast-myofibroblast transformation, Transcorneal penetration

## Abstract

Silver nanoparticles (AgNPs) are a common antimicrobial additive for a variety of applications, including wound care. However, AgNPs often undergo dissolution resulting in release of silver ions, with subsequent toxicity to mammalian cells. The cornea is a primary exposure site to topically administered AgNPs in and around the eye but their impact on corneal wound healing is understudied. Thus, the purpose of this study was to determine *in vitro* toxicity of AgNPs on corneal epithelial cells and fibroblasts as well as their effects on corneal epithelial wound healing utilizing an *in vivo* rabbit model. Non-coated 20 nm sized AgNP (AgNP-20) as well as 1% and 10% silver silica NPs (AgSiO_2_NPs) were tested at concentrations ranging from 0.05–250 μg/mL. Immortalized human corneal epithelial (hTCEpi) cells and primary rabbit corneal fibroblasts (RCFs) were incubated for 24 h with AgNPs and cell viability was tested. Additionally, a round wound healing assay was performed to determine hTCEpi cell migration. Quantitative real-time PCR and western blot analysis was performed to determine α-smooth muscle actin (α-SMA, a myofibroblast marker) mRNA and protein expression, respectively, in RCFs treated with 50 μg/mL of AgNPs. Corneal epithelial wound healing was evaluated with 1%-AgSiO_2_NPs (10 and 250 μg/mL) using an *in vivo* rabbit model. Rabbits were subsequently euthanized, and histologic sections of the enucleated globes were used to determine corneal penetration of 1%-AgSiO_2_NPs with autometallography and hyperspectral darkfield microscopy. Cell viability of both the hTCEpi cells and fibroblasts was significantly decreased by the three AgNPs in a dose dependent manner. Migration of hTCEpi cells was significantly inhibited by the three AgNPs. Alpha-SMA mRNA expression was significantly inhibited with three AgNPs, but only the 1%-AgSiO_2_NPs inhibited protein expression of α-SMA. *In vivo* epithelial wound closure did not significantly differ between groups treated with 10 or 250 μg/mL of 1%-AgSiO_2_NPs or vehicle control. The 1%-AgSiO_2_NPs penetrated throughout all corneal layers and into the anterior chamber in all treated eyes with no histopathological changes observed. In conclusion, the 1%-AgSiO_2_NPs are safe and have potential therapeutic applications through its efficacy of the corneal penetration and reduced scar formation during corneal wound healing.

## 1. Introduction

Silver, including silver sulfadiazine, ionized silver, and metallic silver, has been extensively used in medicine for decades primarily as an antimicrobial additive to prevent infection (Barillo and Marx, 2014). Silver nanoparticles (AgNPs) also demonstrate broad-spectrum antimicrobial activity against bacteria (Sotiriou and Pratsinis, 2010; Santoro et al., 2007; Alarcon et al., 2016), viruses (Galdiero et al., 2011), and fungal organisms (Wright et al., 1999; Xu et al., 2013). Furthermore, AgNPs can improve skin regeneration in patients with infected burns (Jahromi et al., 2018), and many commercial silver-contained wound dressings are available (Mooney et al., 2006). Subsequently, dressings with AgNPs promote healing of skin wounds with improved cosmesis compared with both control (Guthrie et al., 2013) and silver sulfadiazine treatments (Tian et al., 2007).

The antibacterial efficacy of AgNPs results from altering bacterial cell membrane integrity, followed by damage to DNA as well as interfering with cell wall function (Duran et al., 2016). Additionally, AgNPs often undergo dissolution resulting in release of silver ions with a broad range of toxicities, whereby bacteria are more sensitive than mammalian cells (Flores-Lopez et al., 2019; Santoro et al., 2007). The properties of AgNPs can be manipulated particularly with regard to shape (Nguyen et al., 2021a), size (Nguyen et al., 2021b), and functionalization (Luo et al., 2019) to improve antimicrobial efficacy with preserving biocompatibility with the tissue of interest.

The cornea is the outermost transparent layer of the eye comprised of a highly organized trilaminar sandwich with a hydrophobic, multilayered epithelium, a hydrophilic stroma and a hydrophobic, singlelayer endothelium (Last et al., 2012). The corneal epithelium interfaces with the external environment, serving as a barrier against exogenous agents and protects intraocular structures from physical trauma. Specifically, the corneal epithelium limits paracellular entry of foreign materials including many drugs *via* tight junctions between the apical cells (Lakhani et al., 2018). Nanomaterials have been tested as a therapeutic carrier for ophthalmic drugs given that their small size can permit penetration through these junctions, facilitate opening of tight junctions to induce paracellular transport and prolong drug residence time on the ocular surface in the precorneal region (Luo et al., 2020; Jian et al., 2017; Lou et al., 2021).

Each layer of the cornea must be able to heal appropriately following wounding to maintain transparency. Epithelial wound healing of cornea involves cell migration, proliferation and finally adhesion to the underlying basement membrane (Yazdanpanah et al., 2017). When the corneal stroma is wounded, keratocytes are activated and subsequently transformed into fibroblasts and myofibroblasts, termed keratocyte-fibroblast-myofibroblast (KFM) transformation (Myrna et al., 2009; Raghunathan et al., 2017). Extended persistence of the myofibroblasts can lead to corneal fibrosis, also known as stromal haze, which is a leading cause of blindness (Wilson, 2012). There have been limited studies of the impact of AgNPs on corneal epithelial wound healing and KFM transformation.

The purposes of this study were to evaluate the impact of AgNPs on corneal epithelial and stromal cell viability, migration and KFM transformation *in vitro* as well as corneal epithelial wound healing *in vivo*.

## 2. Materials and methods

### 2.1. Characterization of AgNPs and preparation of AgNP suspensions

The 20 nm AgNPs (AgNPs-20), ~8 nm silver supported on silica (3.79% *w*/w of Ag; 1%-AgSiO_2_NPs), and ~ 7 nm silver supported on silica (15.92% w/w of Ag; 10%-AgSiO_2_NPs) were procured, synthesized, and characterized by the Engineered Nanomaterials Coordination Core and part of the Nanotechnology Health Implications Research Consortium at the Harvard T.H. Chan School of Public Health (Beltran-Huarac et al., 2018). Specifically, Ag and AgSiO_2_ nanoparticles (NPs) were synthesized utilizing the Versatile Engineered Nanomaterial Generation System (VENGES) specifically designed for nanotoxicological studies, as presented elsewhere (Demokritou et al., 2010; Tsai et al., 2012). Citrate-capped gold NPs (15 nm; Au) were synthesized following the Turkevich method and characterized by Dong and coworkers (Dong et al., 2020).

Prior to the use of the NPs for this experiment, resuspended NPs were sonicated using a calibrated sonication system (2510R-MT; Branson Ultrasonic Co., Danbury, CT) following the protocol reported previously (DeLoid et al., 2017; Cohen et al., 2018). Briefly, each NP was placed into a 15-mL conical tube and deionized water (DW) added to achieve a final concentration of 2.5 mg/mL. Next, the nanosuspensions were vortexed at high speed for 30 s and sonicated for 3–6 min as determined by the aforementioned protocol for each material (Supplementary Table 3). Following sonication, the stock suspensions were vortexed again for at least 30 s at high speed and diluted with balanced salt solution (BSS; Alcon^®^, Fort Worth, Texas) or culture medias to the final concentration. All diluted suspensions were used immediately after preparation and all preparation procedures were repeated every 24 h. Stability of AgNPs in suspension was tested with dynamic light scattering (DLS). The hydrodynamic diameter and polydispersity index were measured using a DLS instrument (Zetasizer Nano S90, Malvern Instruments Ltd., Malvern, UK) at 1 and 24 h after preparation.

### 2.2. Isolation and cultures of primary stromal cells from rabbit corneas, and culture of immortalized corneal epithelial cells

Primary rabbit corneal fibroblasts (RCFs) were isolated from freshly enucleated young rabbi eyes (Pel-Freez, Rogers, AR) as previously described (Dreier et al., 2013). The isolated cells were cultured in Dulbecco’s modified Eagle’s medium (DMEM) low glucose (MyClone; GE Healthcare Life Sciences, Logan, UT) supplemented with 10% fetal bovine serum (FBS; Atlanta Biologicals, Lawrence, GA) and 1% penicillin-streptomycin-amphotericin B (PSF; Lonza, Walkersville, MD). Cells were incubated for 3 days to assure transition to the fibroblast phenotype and then used between passage 3 to 5 in this study.

Human telomerase reverse transcriptase-immortalized corneal epithelial (hTCEpi) cells, graciously donated by James Jester, PhD (University of California, Irvine), were used between passage 48 to 56. The hTCEpi cells were cultured in growth medium composed of Epilife® (Life Technologies, Carlsbad, CA) supplemented with 1% Epilife Defined Growth Supplement (EDGS®; a proprietary combination of bovine serum albumin, bovine transferrin, hydrocortisone, recombinant human-like growth factor type-1, prostaglandin, and recombinant human epidermal growth factor; Life Technologies) and 1% penicillin-streptomycin-amphotericin B (HyClone^™^ 100×; HyClone) at 37 °C and 5% CO_2_.

### 2.3. Viability assays

The MTT (3-[4,5-dimethylthiazol-2-yl]-2,5 diphenyl tetrazolium bromide) and Calcein-AM (acetoxymethyl ester) assays were used to determine the effects of the AgNPs on hTCEpi cell and RCF viability. The hTCEpi cells and RCFs were plated into 96-well plates at a density of 7000 and 2000 cells per well, respectively, in 100 μL culture medium 24 h prior to treatment to permit attachment. Then, cells were treated for 24 h with the AgNPs-20, 1%-AgSiO_2_NPs and 10%-AgSiO_2_NPs at concentrations between 0.05 and 250 μg/mL in six replicates. Citrate capped gold NPs (AuNPs; 5 μg/mL), and an equal volume of deionized water (DW) to the treatment volume of the NP suspensions were used as negative and vehicle controls, respectively. Saponin (1 mg/mL) was used as positive control for loss of cell viability. For the colorimetric cell viability assay, MTT (0.5 mg/mL) solution was added to each well and incubated for 4 h at 37 °C. The supernatant was carefully aspirated from the wells without disturbing the formazan precipitate. The formazan crystals were dissolved in 50 μL/well with dimethyl sulphoxide (DMSO; Sigma Chemical Co., St. Louis, MO) and mixed thoroughly. The absorbance was measured at 540 nm using a microplate spectrophotometer (Synergy 4; BioTek, Instruments Inc., Winooski, VT).

The Calcein-AM Cell Viability Kit (TREVIGEN®, R&D Systems, Inc. Minneapolis, MN) was used for the fluorometric cell viability assay. Viable hTCEpi cells and RCFs were fluorescently labeled by Calcein-AM (1 μM for 30 min) following a 24 h incubation with one of the aforementioned AgNPs, AuNPs, DW or saponin. The fluorescence intensity was measured with a 490 nm excitation filter and a 520 nm emission filter using a microplate spectrophotometer (Synergy 4).

All viability tests were performed in triplicate; absorbance of NPs or vehicle only without cells at the aforementioned concentrations was also measured as a blank control for both assays. Then, the absorbed values for each concentration of NPs tested with MTT and Calcein-AM assays (NPs only without cells) were subtracted from the original values (NPs with cells) for the final calculation to control for any impact of NPs on the viability measurement; these values were normalized to the vehicle control, and final results were expressed as percent viability relative to the vehicle control.

### 2.4. RNA extraction and quantitative real-time PCR

The RCFs were cultured in 6-well plates for 24 h reaching ~30% confluence before NP treatment. Each well was treated with 50 μg/mL of AgNPs-20, 1%- AgSiO_2_NPs, 10%-AgSiO_2_NPs or DW (vehicle) for 24 h in the presence or absence of TGF-β1 (10 ng/mL) to induce myofibroblast transformation. The 10 ng/mL of TGF- β1 has been previously shown to induce myofibroblast transformation in rabbit fibroblasts (Myrna et al., 2012; Dreier et al., 2013). The RNA was extracted 24 h after TGF-β1 induction using the RNeasy kit (Qiagen, Valencia, CA) following the manufacturer’s protocol. Quantitative real-time PCR (qPCR) was performed with 50 ng of RNA per sample using the Taqman One-Step PCR kit (Life Technologies) and aptamers specific to rabbit α-SMA (*ACTA2*, Oc03399251_m1; Life Technologies) in total volumes of 10 μL per reaction. The reverse transcription reaction was performed in a StepOne qPCR machine (Life Technologies) for 30 min at 50 °C, followed by PCR enzyme activation for 10 min at 95 °C and 40 cycles of 60 °C for 1 min, followed by 95 °C for 15 s. Glyceraldehyde 3-phosphate dehydrogenase (*GAPDH*, Oc03823402_g1) served as a reference. The reactions were run in triplicate, and each experiment was performed at least three times. Unless stated otherwise, gene expression was normalized relative to the expression of mRNA from cells treated with vehicle (DW) without TGF-β1 added to the medium, which was given an arbitrary value of 1.0.

### 2.5. Protein sample preparation and western blot analysis

The RCFs were treated with 50 μg/mL of AgNPs-20, 1%- AgSiO_2_NPs, or 10%-AgSiO_2_NPs in the presence or absence of 10 ng/mL of TGF-β1 for 24 h; the concentrations of AgNP selected were those that demonstrated a significant decrease in cell viability to ~60–70%. After induction, cells were lysed on ice with radioimmunoprecipitation assay (RIPA) buffer containing Halt protease inhibitor cocktail (Thermo Fisher Scientific, Rockford, IL). Insoluble cellular debris was removed by centrifugation at 14,000*g* in a cooled tabletop microcentrifuge for 5 min. The protein concentration was determined using a modified Lowry assay (DC protein assay; Bio-Rad, Hercules, CA) with bovine serum albumin as the standard. The samples were prepared for electrophoresis by incubation with 5× Laemmli buffer at 95 °C for 5 min.

Equivalent amounts of protein (10 μg) were loaded onto a 10% NuPAGE Bis-Tris Pre-Cast gel (Life Technologies). Gel electrophoresis was performed at 140 V for 1 h, followed by transfer to a nitrocellulose membrane (Life Technologies) at 200 mA for 2 h. Successful protein transfer was confirmed by Ponceau S staining. The membrane was blocked for 1 h at RT with SuperBlock blocking buffer (Thermo Fisher Scientific), followed by incubation with primary antibody mouse anti-α-SMA (Sigma-Aldrich Corp.) diluted 1:20,000 in 10% SuperBlock in PBS overnight at 4 °C. The blot was washed three times in PBS with 0.1% Tween-20 (PBS-T) before incubating with peroxidase-conjugated goat anti-mouse antibody (KPL, Gaithersburg, MD) diluted 1:20,000 for 1 h at RT. After washing three times with PBS-T and once with PBS, protein bands of interest were detected using an enhanced chemiluminescent Western blot reagent (ECL plus, GE Healthcare, Pittsgurgh, PA) and an ImageQuant 350 charge-coupled device camera (GE Healthcare). Afterwards, the same blots were used to detect glyceraldehyde 3-phosphate dehydrogenase (GAPDH) as a reference protein. The blot was stripped with Restore^™^ Western Blot Stripping Buffer (Thermo Fisher Sicentific) for 20 min at room temperature and re-probed with anti-GAPDH (mouse anti-GAPDH, catalog number MAB374; EMT Millipore, Billerica, MA) diluted 1:2000 in blocking buffer at 37 °C for 1 h. Densitometry analyses were done with ImageJ software (NIH). The band densities of αSMA were normalized to the values of GAPDH. Western blot analysis was performed on samples from at least three independent experiments.

### 2.6. In vitro epithelial cell migration assay

Cell migration of hTCEpi cells *in vitro* was measured using the Oris^™^ 96-well cell migration assay kit (Platypus Technologies, Madison, WI) following the manufacturer’s instructions. In brief, 7 × 10^4^ cells/mL of hTCEpi cells in 100 μL of culture medium were seeded in each well of the 96-well plate, and were allowed to attach for 24 h at 37 °C. Once the cells formed a confluent monolayer, the stoppers were removed to permit cells to migrate into the detection zone. Concomitantly, the cells were treated with the AgNPs-20, 1%- or 10%-AgSiO_2_NPs with selected concentration for each material based on the viability assay results; five concentrations of each NP were utilized including the lowest concentration that significantly decreased cell viability. The hTCEpi cells were treated with DW or cytochalasin D (1 μg/mL) as vehicle or positive controls, respectively. Twenty-four h after initiation of the migration, cells were fixed with 4% paraformaldehyde in PBS (Thermo Fisher Scientific Chemicals Inc., Waltham, MA) for 30 min. Nuclei were stained for 10 min at room temperature (RT) with 4′,6-diamidino-2-phenylindole (DAPI; BioGenex, San Ramon, CA) 1:5000 in PBS. Cells were imaged immediately after staining using a fluorescence microscope with 5× objective (Axiovert 200 M; Carl Zeiss, Jena, Germany) or 4× objective (BZ-X800; Keyence Co., Osaka, Japan). The area devoid of cells was measured using ImageJ analysis software (ver 1.51j8; National Institutes of Health, Bethesda, MD). Data were expressed as relative percent migration compared with the vehicle control (DW).

### 2.7. Animals

Eighteen New Zealand White rabbits (Charles River laboratories, Wilmingon, MA), 6 males and 12 females, were utilized in this study with a mean age of 4.0 ± 0.0 months and body weight of 3.25 ± 0.86 kg. Animals were divided into three groups with the following treatments: (1) BSS (vehicle control; *n* = 6), (2) 10 μg/mL of 1%-AgSiO_2_NPs suspension (n = 6) as a low concentration group, and (3) 250 μg/mL of 1%- AgSiO_2_NPs suspension (n = 6) as a high concentration group. The study design was approved by the Institutional Animal Care and Use Committee of the University of California, Davis (IACUC #19691) and performed in compliance with the Association of Research in Vision and Ophthalmology (ARVO) statement for the use of animals in ophthalmic and vision research. A complete ophthalmic examination and advanced ocular imaging were performed prior to inclusion in the study; only animals free of ocular disease were used.

### 2.8. Test article preparation and treatments

For the *in vivo* study, the highest tolerated dose (~2.5 μg/mL) of 1%- AgSiO_2_NPs was selected based on the *in vitro* toxicity and migration assays and multiplied by 100-fold (250 μg/mL) for use as a high-dose topical treatment to be administered six times daily while considering the following factors: (1) immediate dilution of the 40 μL NP suspension by the tear film on the ocular surface, and (2) exposure time on the corneal surface that the NP suspension will likely reside on the ocular surface after topical application. The 10 μg/mL of 1%-AgSiO_2_NPs was selected for the low concentration group from the lowest *in vitro* concentration with an effect given the aforementioned factors.

All treatments were prepared fresh daily prior to the first treatment. For the preparation, a stock solution of the 1%-AgSiO_2_NPs concentration of 10 mg/mL in DW was vigorously vortexed for 30 s and subsequently sonicated for 3.2 min per 1 mL of the 1%-AgSiO_2_NPs stock suspension. Following sonication, the 1%-AgSiO_2_NPs stock suspension was vortexed again for 30 s before use, and then the 1%-AgSiO_2_NPs stock suspension was diluted in BSS to final concentrations of 10 or 250 μg/mL and aliquoted into 1.5-mL microcentrifuge tubes. The aliquoted 1%-AgSiO_2_NP suspensions and BSS (vehicle) were stored at 4 °C until use and vortexed at least 30 s immediately prior to treatment. Forty μL of treatment or vehicle controls were given six times daily to both eyes (oculus uterque, OU).

### 2.9. Epithelial debridement

Rabbits were pre-medicated with midazolam (0.7 mg/kg) by intramuscular injection (IM) and hydromorphone (0.1 mg/kg, IM) followed by ketamine (10–30 mg/kg, IM) for induction and maintenance of anesthesia. Then, the surgical area was prepped with 0.2% povidone iodine and saline. The cornea of the right eye (oculus dexter, OD) was anesthetized with 0.5% proparacaine hydrochloride ophthalmic solution (Alcon laboratories, Inc., Fort Worth, TX) and the corneal epithelium was marked in the central 8-mm cornea with a corneal trephine. The marked area was debrided using a blunt spatula (Excimer Spatula; Beaver-Visitec, Waltham, MA) and confirmed by retention of fluorescein sodium (NaFL) stain (BIO GLOTM; 1 mg, HUB Pharmaceuticals, LLC., Rancho Cucamonga, CA); the stain was imaged with a digital camera (Nikon D300, Nikon co., Tokyo, Japan; flash 1/4, iso 250, F11; with cobalt blue filters [Blue-AWB, Nikon] flash coverings and a yellow lens filter [HMC 62 mm Y[K2], HOYA]) to establish wound area at baseline. Atropine sulfate 1% (Akorn, Inc., Lake Forest, IL) and ofloxacin 0.3% (Alcon, Hunengerg, Switzerland) ophthalmic solutions were administered OD following the epithelial debridement five minutes apart from each treatment, then, five minutes later, artificial tear ointment (Rugby Laboratories, Inc., Duluth, GA) was applied in both eyes to prevent the corneal dessication during anesthetic recovery. Buprenorphine (0.03–0.06 mg/kg, IM) was administered to provide analgesia. All animals were monitored every 10 min until they return to their normal upright body positions.

### 2.10. Ocular exam scoring, imaging and image analysis

A complete ophthalmic examination was performed prior to and daily after surgery until the integrity of the corneal epithelium had been re-established in the vehicle control group as determined by the absence of fluorescein stain retention. A semiquantitative preclinical ocular toxicology scoring (SPOTS) system (Eaton et al., 2017) was used to assess the eyes using a hand-held slit lamp biomicroscope (SL-15; Kowa Company, Ltd., Tokyo, Japan). Rebound tonometry was performed to measure intraocular pressure (TonoVet; Icare, Helsinki, Finland). The NaFL stain was applied twice daily after the surgery and photographed to assess epithelial wound area. All images were analyzed using ImageJ software (ver 1.51j8; NIH). Each photograph was analyzed to calculate the percentage wound closure (tracing the border of the fluorescein-stained area). The percent remaining wound area at each time point was calculated from the photographic images using the following equation:

%Woundarea=WoundareaondayX/Woundareaonday0×100

### 2.11. Tissue harvest, processing, and staining

Rabbits were euthanized with pentobarbital (200 mg/kg, IV) at 4 days post-surgery when the corneal epithelial wound was completely healed in the control group. The enucleated eyes were fixed in 10% neutral buffered formalin and underwent routine paraffin processing. The samples sectioned 5-μm thick at parasagittal plain and stained with hematoxylin and eosin (H&E, Fisher Scientific, CA) prior to evaluation with light microscopy.

Autometallography was performed on the 5-μm thick paraffin embedded parasagittal sections to detect penetration of the 1%-AgSiO_2_NPs using the Silver Enhancing Kit (BB international., Crumlin, UK) following manufacturers’ instructions. Briefly, paraffin was dissolved in xylene for 10 min twice and samples were rehydrated using serial dilution of ethanol. The samples were then rinsed in water for 5 min twice and slides gently tapped on a paper towel to remove excess water before applying enhancer. After mixing equal quantities of initiator and enhancer together, and apply the solution as a drop, to the samples in normal RT. After 20 min for enhancement, the slides were washed with purified water for 2–3 min. The 1% toluidine blue was applied for 5 min for the counter stain, then the slides washed again with purified water. The sample were then dehydrated using 70, 95 and 100% ethanol and xylene for 1 min per step and a coverslip was applied with a mounting media (VectaMount^™^ Permanent Mounting Medium; Vector Laboratories, Inc., Burlingame, CA). Images were captured with a bright field microscope (Nikon Eclipse 800, equipped with a Q Imaging Exi Aqua camera and Chroma Technology filter sets). In addition, deparaffinized 5-μm thick parasagittal sections were also used for the hyperspectral darkfield-based microscopy using a high signal-to-noise, darkfield-based illumination on Olympus BX-41 microscope (Cytoviva, Auburn, AL) to determine transcorneal penetration of the 1%-AgSiO_2_NPs.

### 2.12. Statistical analysis

Data were presented as mean ± standard deviation (SD); statistical analysis was performed with GraphPad Prism 7.03 (GraphPad Software Inc., San Diego, CA). All data sets were compared with one-way or two-way analysis of variance (ANOVA) as indicated. When variability between data sets were determined to be significant, a Tukey’s multiple comparisons test was used to compare them. Values of *P* < .05 were considered statistically significant.

## 3. Results

### 3.1. Physicochemical characterization of the AgNPs

Detailed characterization of the AgNPs-20, 1%- and 10%-AgSiO_2_NPs utilized in this study has been previously published by Beltran-Huarac and coworkers (Beltran-Huarac et al., 2018). In summary, all the AgNPs showed a high degree of chemical purity (Supplementary Table 1) and stoichiometry, and low content of carbon residuals (Supplementary Table 2). Additionally, the AgNPs were sterile with no bacterial or endotoxin contamination (Supplementary Table 2).

### 3.2. The AgNPs did not agglomerate in suspension up to 24 h

The colloidal properties of the three AgNP suspensions in Epilife®, DMEM media or BSS were measured by DLS. The mean hydrodynamic diameter of the AgNPs, 1%-AgSiO_2_NPs and 10%-AgSiO_2_NPs in suspensions are shown in Supplementary Table 3, along with <0.7 for the polydispersity indexes. The hydrodynamic diameters of the AgNPs and 1%-AgSiO_2_NPs suspensions in three different media showed no significant differences between 1- and 24-h post-suspension, indicating particle size distribution did not change significantly over 24 h in the suspension. Hydrodynamic diameter significantly decreased from 668.3 ± 242.2 nm to 294.3 ± 38.3 nm at 1- and 24-h, respectively for the 10%-AgSiO_2_NPs suspended in Epilife® (*P* < .001).

### 3.3. The AgNPs significantly influenced cell viability at concentrations ≥12.5 μg/mL

**Fig. 1.**
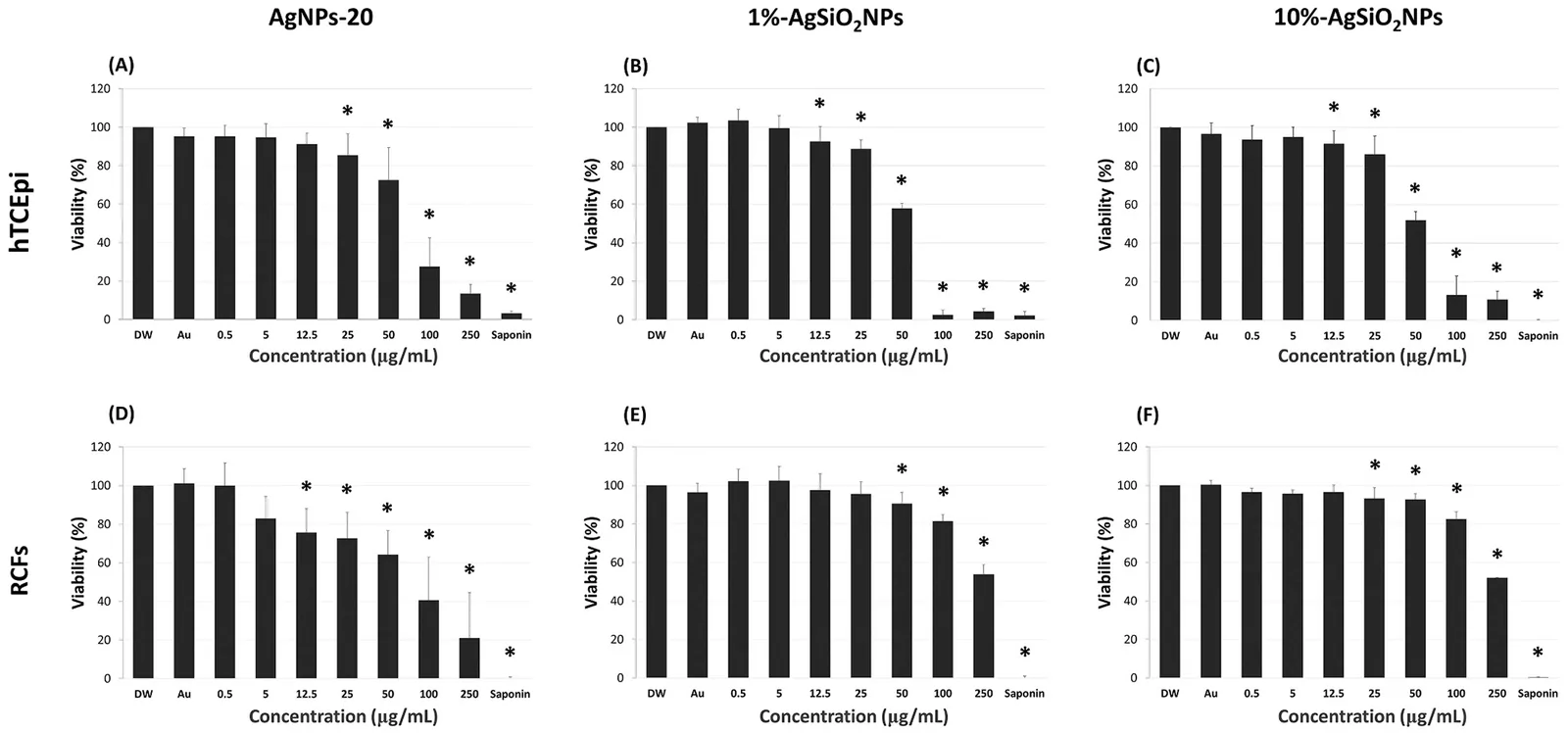
Dose dependent decrease in cell viability observed following treatment with three silver nanoparticles. MTT assay revealed similar cell viability results for the hTCEpi cells (A-C) and RCFs (D–F). AuNPs (5 μg/mL), DW and saponin (1 mg/mL) were used as negative, vehicle and positive controls, respectively. The data represent mean ± standard deviation (SD), *n* ≥ 3. Error bars represent the SD and asterisks indicate significant differences compared with the vehicle controls. *P < .05, one-way ANOVA followed by Tukey’s multiple comparisons. DW, deionized water; Au, 15 nm citrate capped (in suspension) gold nanoparticles; AgNPs-20, 20 nm silver nanoparticles; 1%-AgSiO_2_NPs, ~8 nm silver supported on silica (3.79% *w*/w of Ag); 10%- AgSiO_2_NPs, ~7 nm silver supported on silica (15.92% *w*/w of Ag).

Both MTT (Fig. 1) and Calcein-AM assays (Supplementary Fig. 1) showed similar toxicity results for the three types of AgNPs. The AgNPs-20 significantly reduced cell viability at ≥25 μg/mL for the hTCEpi cells (*P* < .001) and ≥ 12.5 μg/mL for the RCFs (*P* < .001) in both assays. The 1%-AgSiO_2_NPs and 10%-AgSiO_2_NPs also significantly reduced viability of the hTCEpi cells at concentrations ranging from 12.5 to 250 μg/mL (*P <* .001). With the two AgSiO_2_NPs, hTCEpi cell viability at >100 μg/mL was dramatically decreased to <20% compared with vehicle controls. Viability of the RCFs was significantly reduced at ≥50 μg/mL of 1%-AgSiO_2_NPs (*P* < .02) and ≥ 25 μg/mL of 10%-AgSiO_2_NPs (*P* < .01).

### 3.4. 1%-AgSiO_2_NPs significantly inhibited the KFM transformation

**Fig. 2.**
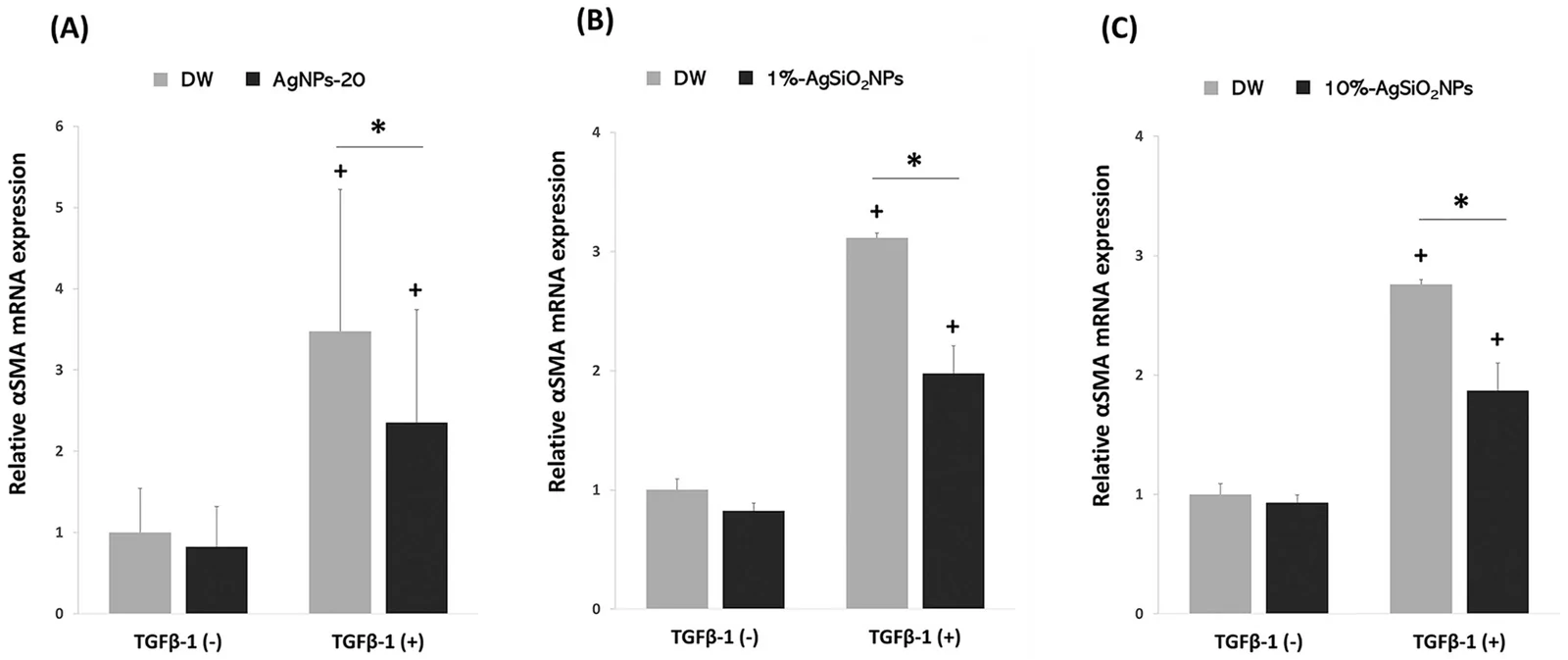
Treatment with all three AgNPs significantly inhibited alpha-SMA mRNA expression in RCFs after treatment. Gene expression of αSMA treated with 50 μg/mL of AgNPs-20 (A), 1%-AgSiO_2_NPs (B) and 10%-AgSiO_2_NPs (C) was analyzed by quantitative real-time PCR with normalization against GAPDH. DW was used as vehicle controls. The data represent mean ± standard deviation (SD), n ≥ 3. Error bars represent the SD. *P < .05, indicating significant differences in between DW *versus* AgNPs-treated groups for the two-way ANOVA followed by Tukey’s multiple comparisons.; +*P* < .05, indicating significant differences in the presence or absence of TGFβ-1 treatments within the same treatment group for the two-way ANOVA followed by Tukey’s multiple comparisons. DW, deionized water; AgNPs-20, 20 nm silver nanoparticles; 1%-AgSiO_2_NPs, ~8 nm silver supported on silica (3.79% *w*/w of Ag); 10%- AgSiO_2_NPs, ~7 nm silver supported on silica (15.92% w/w of Ag).

**Fig. 3.**
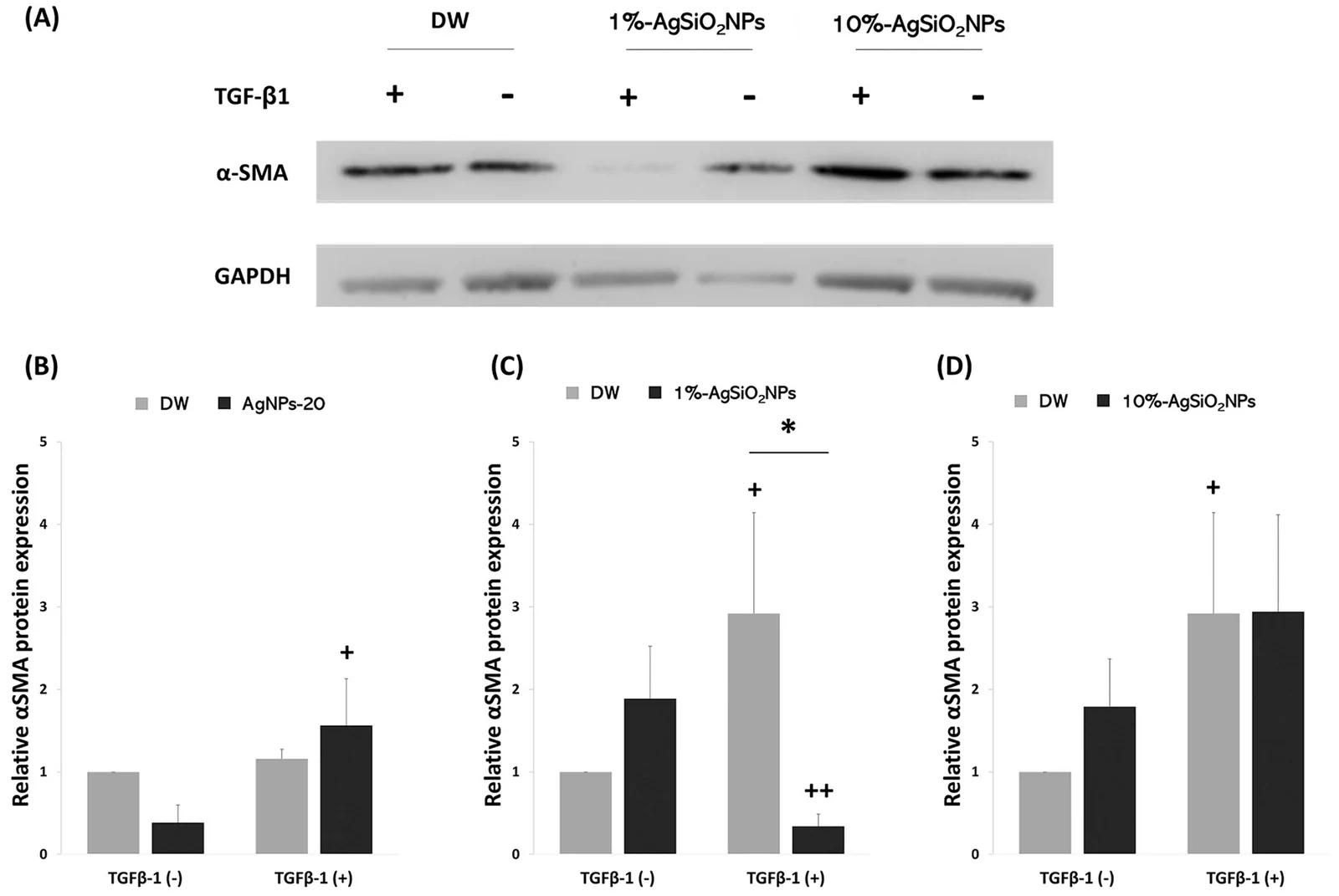
Significantly inhibited alpha-SMA protein expression in RCFs after treatment with 1%-AgSiO_2_NPs. (A) Representative western blot image of the protein expression. Relative αSMA protein expression after treatment with AgNPs-20 (B), 1%-AgSiO_2_NPs (C) and 10%-AgSiO_2_NPs (D) in the presence or absence of TGFβ-1. Relative αSMA protein expression was determined by calculating the densitometry ratio αSMA to GAPDH protein bands and normalizing each ratio with the vehicle control in the absence of TGFβ-1 treatment. DW was used as vehicle controls. The data represent mean ± standard deviation (SD), n ≥ 3. Error bars represent the SD. *P < .05, indicating significant differences in between DW *versus* AgNPs-treated group for the two-way ANOVA followed by Tukey’s multiple comparisons.; +*P* < .05, indicating significant differences in the presence or absence of TGFβ-1 treatments within the same treatment group for the two-way ANOVA followed by Tukey’s multiple comparisons. DW, deionized water; AgNPs-20, 20 nm silver nanoparticles; 1%-AgSiO_2_NPs, ~8 nm silver supported on silica (3.79% w/w of Ag); 10%- AgSiO_2_NPs, ~7 nm silver supported on silica (15.92% w/w of Ag).

As expected, treatment with 10 ng/mL TGF-β1 induced the expression of α-SMA mRNA in RCFs with 24 h of incubation, indicating the transformation of RCFs to myofibroblasts. In RCFs treated with 50 μg/mL of AgNPs-20, 1% and 10%-AgSiO_2_NPs in the present of TGF-β1, α-SMA mRNA expression was significantly decreased compared to vehicle control (*P* = .020, 0.008, and 0.049, respectively) (Fig. 2). However, without added TGF-β1, no statistically significant differences were observed with AgNPs treatment (*P* > .05). We detected significantly reduced amounts of α-SMA protein in RCFs cultured with 1%-AgSiO_2_NPs in the present of TGF-β1 (*P* < .001) (Fig. 3). However, no statistical differences were identified for the 10%-AgSiO_2_NPs or AgNPs-20 treatment. Thus, KFM transformation was only significantly inhibited by the 1%-AgSiO_2_NPs when considering protein expression.

### 3.5. Cell migration was significantly decreased by the AgNPs in dose dependent manner

**Fig. 4.**
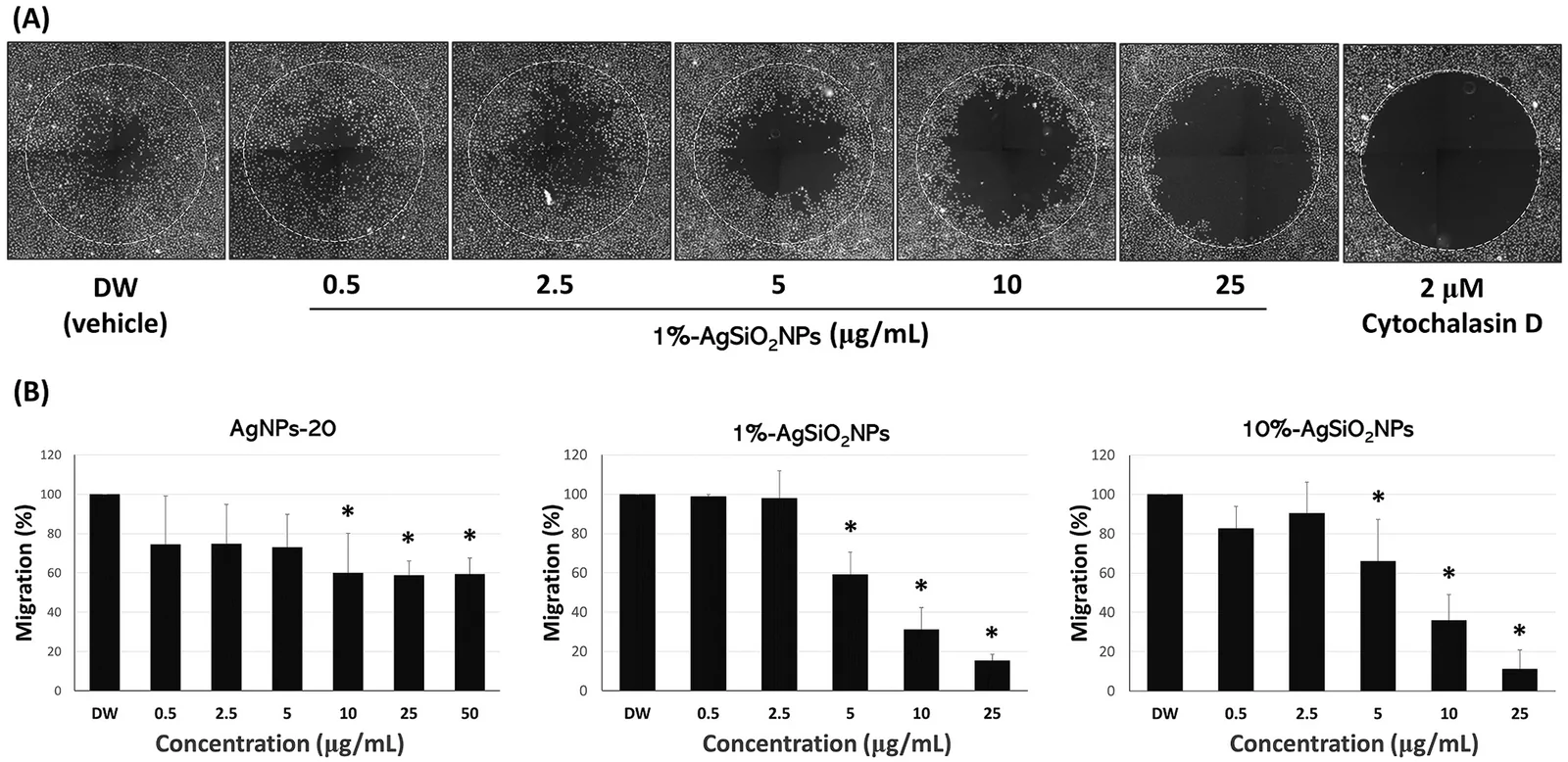
Corneal epithelial cell migration *in vitro* was significantly reduced by three AgNPs in dose dependent manner. The Oris^™^ 96-well cell migration assay kit was used to assess cell migration *in vitro*. (A) Representative images of migration assay of hTCEpi cells treating with 0.5–25 μg/mL of 1%-AgSiO_2_NPs. (B) Compared with AgNPs-20, 1%- and 10%-AgSiO_2_NPs showed more potent at inhibiting cell migration in concentration higher than 5 μg/mL. DW and cytochalasin D were used as vehicle and positive controls, respectively. The data represent mean ± standard deviation (SD), n ≥ 3. Error bars represent the SD. *P < .05, one-way ANOVA followed by Tukey’s multiple comparisons test was performed to compare with the vehicle (DW) controls. DW, deionized water; AgNPs-20, 20 nm silver nanoparticles; 1%-AgSiO_2_NPs, ~8 nm silver supported on silica (3.79% w/w of Ag); 10%- AgSiO_2_NPs, ~7 nm silver supported on silica (15.92% w/w of Ag).

Migration of hTCEpi cells was significantly delayed for AgNPs-20 at ≥10 μg/mL (*P* < .001) and 1%- and 10%-AgSiO_2_NPs at ≥5 at μg/mL (*P* < .001) (Fig. 4). The 1%- and 10%-AgSiO_2_NPs inhibited cell migration more severely at lower concentrations and demonstrated more cytotoxicity than the AgNPs-20. Although the aforementioned hTCEpi cell viability and migration assay results were similar for the 1%-AgSiO_2_NPs and 10%-AgSiO_2_NPs treatments, the 1%-AgSiO_2_NPs were more stable in hydrodynamic size than the 10%-AgSiO_2_NPs (Supplementary Table 3). Therefore, we selected the 1%-AgSiO_2_NPs to test its impact further on corneal epithelial wound healing *in vivo*.

### 3.6. No significant effects were observed in vivo corneal epithelial wound healing by topical treatments of 1%-AgSiO_2_NPs suspension

**Fig. 5.**
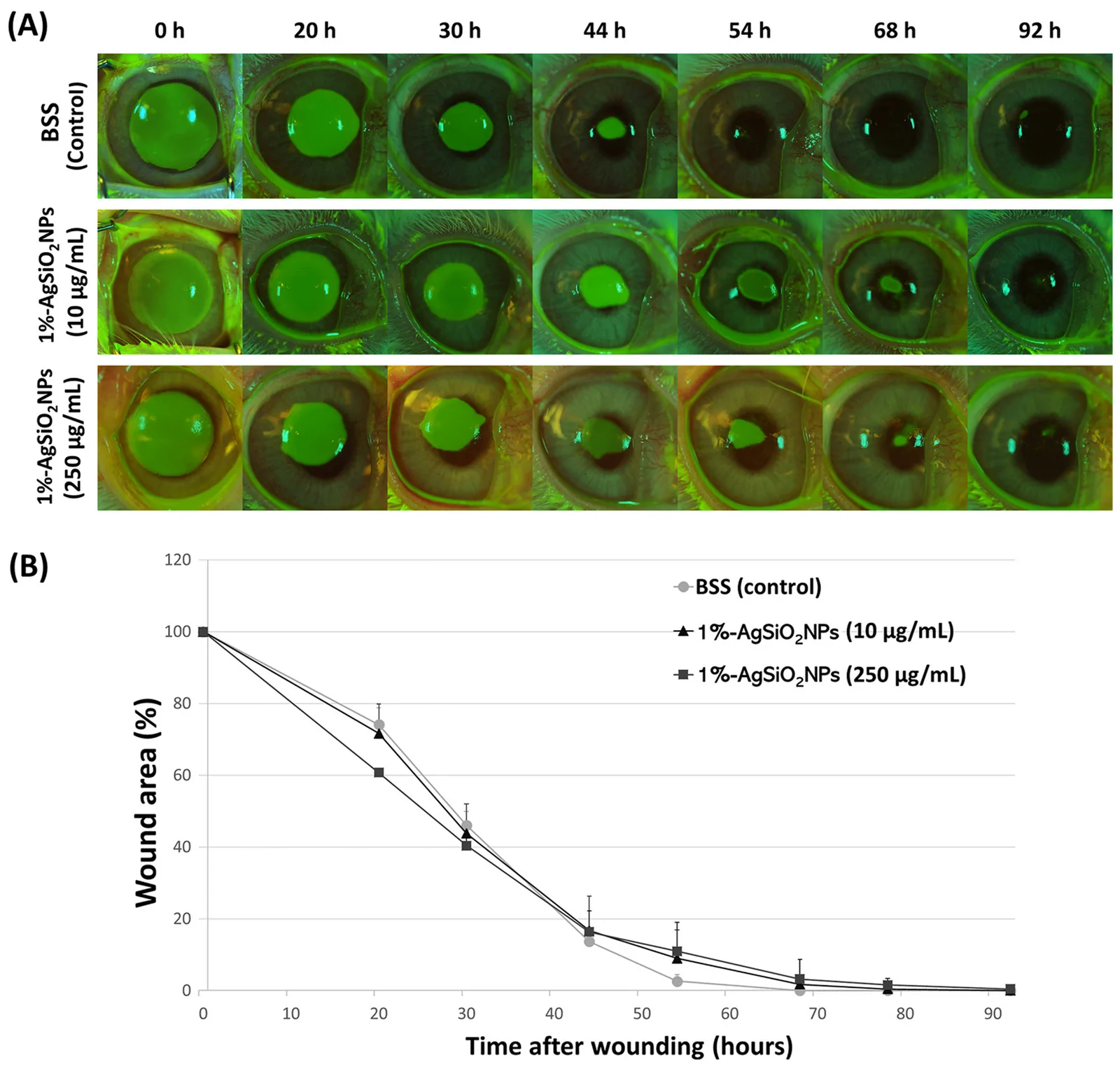
Topical low- and high-dose of 1%-AgSiO_2_NPs showed no significant delay in epithelial wound repair in an *in vivo* rabbit epithelial debridement model. (A) Representative images of corneal epithelial wound healing in rabbits treated with topical 10 and 250 μg/mL 1%-AgSiO_2_NPs or BSS, as a vehicle control, six times daily. (B) No significant differences of corneal epithelial wound healing rates were identified between groups at all time points. The data represent mean ± standard deviation (SD), *n* = 6 in each group. Error bars represent the SD. Repeated measures two-way ANOVA followed by Tukey’s multiple comparisons. BSS, balanced salt solution; 1%-AgSiO_2_NPs, ~8 nm silver supported on silica (3.79% w/w of Ag).

The corneal epithelial wound healing rates, with low and high concentrations of 1%-AgSiO_2_NPs and vehicle (BSS) control treatment conditions, were measured using digital images of the sodium fluorescein staining with all rabbits healed at 92 h post-wounding (Fig. 5A). Compared with BSS control, the 10 and 250 μg/mL 1%-AgSiO_2_NPs treatment group showed no significant differences in wound healing rates at all the time points (*P* = .53 and 0.77, respectively) (Fig. 5B). In the wounded eyes, conjunctival hyperemia and swelling, discharge, corneal opacity and iris hyperemia were transiently observed until the corneal epithelial wounds healed (Supplementary Fig. 2). However, there were no significant differences between groups for all scoring criteria and all abnormal findings resolved with complete closure of the epithelial wound. In non-wounded eyes, no remarkable clinical findings were observed with both low and high concentrations of 1%-AgSiO_2_NPs treated groups as well as vehicle control during all observed timepoints.

### 3.7. 1%-AgSiO_2_NPs penetrated all corneal layers without histopathological changes

**Fig. 6.**
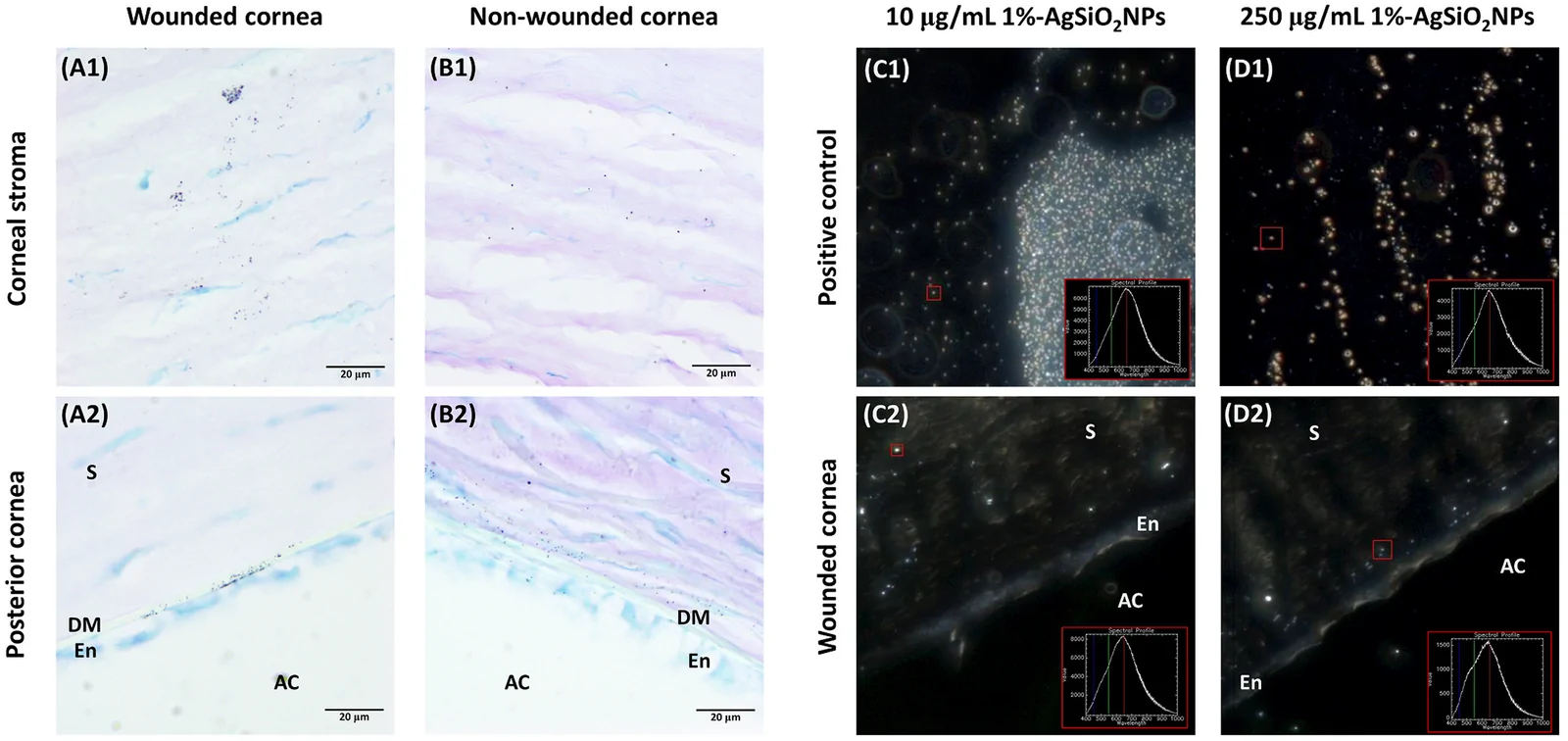
The 1%-AgSiO_2_NPs penetrated all corneal layers following topical treatment in both wounded and unwounded eyes using autometallography (A and B) and hyperspectral darkfield microscopy (C and D). Bright field microscopy showed the stained 1%-AgSiO_2_NPs (black arrows) by autometallography in the wounded (A1 and 2) and non-wounded corneas (B1 and 2) with treatments of 10 μg/mL 1%-AgSiO_2_NPs. The counter staining was performed with 1% toluidine blue. Dark field hyperspectral microscopy showed 1%-AgSiO_2_NPs (white arrows) in the wounded cornea with both 10 (C2) and 250 μg/mL treated corneas (D2). C1 and D1 shows positive controls of the 1%-AgSiO_2_NPs. S, stroma; DM, Descemet’s membrane; En, endothelium; AC, anterior chamber; 1%-AgSiO_2_NPs, ~8 nm silver supported on silica (3.79% w/w of Ag). (For interpretation of the references to colour in this figure legend, the reader is referred to the web version of this article.)

Autometallography and hyperspectral darkfield-based microscopy were performed to determine transcorneal penetration of 1%-AgSiO_2_NPs when topically treated on the wounded cornea (Fig. 6). The results indicated that the 1%-AgSiO_2_NPs were observed in all corneal layers including corneal epithelium, stroma, and endothelium in both low- and high-concentration groups. No histopathological abnormalities, including inflammation, were observed in any tested eyes (data not shown). The 1%-AgSiO_2_NPs were also observed in both wounded and non-wounded eyes. Additionally, a few particles of 1%-AgSiO_2_NPs were also observed in the anterior chamber and iris stroma (Supplementary Fig. 3). The amounts of penetrated particles did not vary with wounding or concentration of the 1%-AgSiO_2_NPs.

## 4. Discussion

In this study, we documented *in vitro* toxicity of AgNPs-20, 1%- and 10%-AgSiO_2_NPs to corneal epithelial cells and fibroblasts as measured by cell viability. All three AgNPs showed significantly reduced viability in both corneal cell types at concentrations greater than 12.5 μg/mL. The α-SMA mRNA expression of RCFs treated with 1%- and 10%-AgSiO_2_NPs at 50 μg/mL was significantly decreased compared to vehicle controls but only the 1%- AgSiO_2_NPs significantly reduced α-SMA protein expression in RCFs. These results indicate that the 1%-AgSiO_2_NPs significantly decreased KFM transformation *in vitro* in RCFs. The 1%-AgSiO_2_NPs at ≥5 μg/mL significantly delayed *in vitro* migration of hTCEpi cells more markedly than the AgNPs-20. However, this alteration in hTCEpi migration was not apparent *in vivo* as topical treatment 1%-AgSiO_2_NPs at 10 and 250 μg/mL resulted in NP residence in all corneal layers without impacting corneal epithelial wound healing in an *in vivo* rabbit model. Collectively, these data suggest that 1%-AgSiO_2_NPs exhibit good corneal penetration, are safe in wounded and unwounded corneas, and may have therapeutic potential to decrease corneal scarring *in vivo*.

Metal-based nanomaterials can exhibit toxicity, even if the same bulk form metal is relatively safe, due to their physicochemical properties including the high surface area to volume ratio, increased generation of reactive oxygen species, aggregation behavior, and/or abundant dissolution of metal ions (Schrand et al., 2010). Metallic NPs have demonstrated variable *in vitro* and *in vivo* toxicity to the cornea, but AgNPs have not been examined in these contexts (Zhou et al., 2014; Kim et al., 2020). In human macrophages, 1%- and 10%-AgSiO_2_NPs showed moderate *in vitro* toxicity amongst twenty metallic NPs tested but were less toxic than zinc oxide (ZnO), vanadium pentoxide (V_2_O_5_), and copper oxide NPs (Zhang et al., 2020). Consistent with these findings, *in vitro* toxicity of the 1%- and 10%-AgSiO_2_NPs as well as AgNPs-20 in this study were moderate to severe but less toxic than ZnO and V_2_O_5_ NPs to hTCEpi cells in a previous study from our lab (Kim et al., 2020). By contrast, 40 nm AgNPs demonstrated minimal cytotoxicity at concentrations ≤6 μM in human corneal epithelial cells (Santoro et al., 2007). Similar, no effects on cell proliferation were observed in human corneal epithelial cells plating on hydrogels coated with ~500 μM of 100 nm AgNPs (Alarcon et al., 2016). In the present study, AgNPs with a diameter of <20 nm were used suggesting that the observed differences in cytotoxicity may be due to size of the AgNPs. Consistent with this theory are observations that AgNPs were also cytotoxic to RCFs in the present study as well as human dermal fibroblasts at 4.7 nm in a previous study by generating oxidative stress (Avalos et al., 2016).

The AgNPs delayed *in vitro* migration of the corneal epithelial cells, but not *in vivo* wound healing. Metal oxide nanomaterials can impede cell migration by changing cellular mechanical properties and force generation (Ali et al., 2017) or altering microtubule remodeling through the production of reactive oxygen species (Apopa et al., 2009). Metal oxide NPs, in particular ZnO, decreased corneal epithelial cell migration *in vitro* and *in vivo* (Zhou et al., 2014; Kim et al., 2020). However, the present study revealed no delay in corneal epithelial wound healing *in vivo* at a dose of 250 μg/mL for the 1%-AgSiO_2_NPs, a 100-times greater concentration than the highest tolerated dose *in vitro*. The discrepancy between the *in vitro* and *in vivo* results in the current study could be explained by both the dilutional effect and the protein binding properties of small molecules within the tear film (Duran et al., 2015). Duan and co-workers demonstrated that the 1%-AgSiO_2_NPs dynamically bound with lysozyme and lactalbumin to form a unique protein corona (Duan et al., 2020). Therefore, topically applied 1%-AgSiO_2_NPs may bind to tear film proteins thus altering their physicochemical properties and biological outcomes.

In dermal wound healing studies, AgNPs are known to promote wound healing through their antibacterial properties and by modulating cytokines to decrease inflammation (Tian et al., 2007). Liu and co-workers reported differential responses of keratinocytes and fibroblasts to AgNPs during dermal wound healing with the AgNPs accelerating wound closure by increasing keratinocyte proliferation and migration, as well as increasing differentiation of fibroblasts into myofibroblasts and thus increasing wound contraction (Liu et al., 2010). In the cornea, myofibroblasts are initially critical for stromal wound healing but persistence within the cornea is associated with scarring and loss of corneal transparency (Raghunathan et al., 2017). In the present study, the 1%-AgSiO_2_NPs decreased α-SMA mRNA and protein expression. These results combined with the observation that the NPs penetrate intact corneal epithelium well and are safe suggests that the 1%-AgSiO_2_NPs may have therapeutic potential to decrease corneal haze development during wound healing. Similar to our findings, Butler and colleagues reported AgNP treated eyes had fewer α-SMA positive myofibroblasts at the conjunctiva using an *in vivo* rabbit filtration surgery model when compared with mitomycin C treatment (Butler et al., 2013). In aggregate, AgNPs may have application for corneal haze and other numerous ocular applications that feature fibrosis.

Transcorneal drug delivery is challenging due to the specific corneal barriers and tear drainage. Various type of NPs has been investigated as drug carriers for improving the penetration to the anterior segment of the eye (Janagam et al., 2017; Jian et al., 2017). Even though corneal penetration and the antimicrobial effects of AgNPs have been studied *in vitro* (Xu et al., 2013), information regarding *in vivo* corneal residence of AgNPs is lacking. Herein, we demonstrated that topically applied 1%-AgSiO_2_NPs (~10 nm average Ag particle diameter) penetrated through all the corneal layers in wounded and unwounded rabbit corneas *in vivo* with no obvious differences, indicating that the NPs were able to permeate an intact corneal epithelium. In a 3D *ex vivo* skin wound model, 20 nm AgNPs did not permeate into the deep dermis, even though 85% of the wounded dermal surface area was covered with AgNPs (Touloumes et al., 2020). Differences in NP tissue penetration between our study and others can be attributed to multiple factors including: size and intrinsic properties of the AgNPs tested, tissue specific differences in between the cornea and skin, and differences between *in vivo* and *ex vivo* environments. In addition, AgSiO_2_NPs significantly increased the permeability coefficient of intestinal epithelium by disrupting tight junction integrity (Mortensen et al., 2020). Furthermore, the AgNPs also altered epithelial basement membrane integrity in *ex vivo* 3D growth models of lung, esophageal, breast and colorectal epithelium (Martin et al., 2019). In aggregate, these studies suggest that AgSiO_2_NPs may have penetrated the corneal epithelium by increasing its permeability. However, the 1%-AgSiO_2_NPs showed no ocular irritation, inflammation, or spontaneous corneal erosions in our *in vivo* study consistent with a previous publication that single topical application of 10 nm colloidal AgNPs demonstrated no toxicity using the acute eye irritation test in normal rabbits (Kim et al., 2013).

In conclusion, AgNPs showed moderate *in vitro* toxicity to corneal epithelial cells and fibroblasts, however, low and high concentrations of 1%-AgSiO_2_NPs showed no alteration in corneal epithelial wound healing and penetrated through all the corneal layers without causing histopathological changes *in vivo*. Moreover, 1%-AgSiO_2_NPs significantly reduced KFM transformation *in vitro* suggesting that it may mitigate scar formation during corneal wound healing. Thus, 1%-AgSiO_2_NPs are safe and efficiently delivered to the cornea and have potential therapeutic applications.

## Supplementary Material

Supplementary data

## Appendix A. Supplementary data

Supplementary data to this article can be found online at https://doi.org/10.1016/j.impact.2021.100352.
