# Supplementary material for: Transcorneal delivery of topically applied silver nanoparticles does not delay epithelial wound healing
Source: NanoImpact. Author manuscript; Available in PMC 2026 Sep 1. (PMC13531338; doi:10.1016/j.impact.2021.100352)

Supplementary Table 1: Summary of physical properties of 1%-$AgSiO_2$NPs, 10%-$AgSiO_2$NPs, and AgNPs-20.

		1%-$AgSiO_2$NPs	10%-$AgSiO_2$NPs	AgNPs-20
**Primary Particle Density**	**ρ** (g/cm^3)	3.2427 ± 0.1376	3.5765 ± 0.2540	8.339 ± 0.398
**Primary Particle Size**	**SSA** (m^2/g)	359.8 ± 14.4	250.1 ± 10.0	31.2 ± 0.5
	d_{BET} (nm)	Ag: 9.3<br>SiO_2: 6.24 ± 0.25	Ag: 6.4<br>SiO_2: 9.17 ± 0.37	18.3 ± 0.3
	$d_{est.}$ (nm)* – Ag	8.17	6.38	-
	d_{TEM} (nm) – Ag	10.64 ± 7.10	7.87 ± 4.29	25.59 ± 6.54
	d_{XRD} (nm) – Ag	7.90	5.80	12.3
**Shape Factors**	**aspect ratio**	1.185 ± 0.166	1.123 ± 0.144	1.185 ± 0.166
	**circularity**	0.922 ± 0.137	0.972 ± 0.086	0.922 ± 0.137
	**roundness**	0.859 ± 0.107	0.889 ± 0.107	0.846 ± 0.096
**Crystal Structure**	**crystallinity** (%)	90.8	86.7	64.8
**Ag Content** (ICP-MS)	**% Ag** (w/w %)	3.79 ± 0.08	15.92 ± 0.25	-

ρ, density of primary particle; SSA, specific surface area as measured by nitrogen adsorption/Brunauer-Emmett-Teller (BET) method; d_{BET}, d_{TEM}, and d_{XRD}, primary particle size as measured by BET, transmission electron microscopy, and XRD, respectively. $d_{est.}$, estimated particle size. *The BET Diameter is calculated based on the mass ratio of silver to the mass of the composite measured via inductively-coupled plasma mass spectrometry, the silver transmission electron microscopy diameter, the shape factors, and the assumption of uniform spherical particles. These data have been previously published by Beltran-Huarac and coauthors (Beltran-Huarac et al., 2018).

Supplementary Table 2: Summary of chemical and biological properties of 1%-$AgSiO_2$NPs, 10%-$AgSiO_2$NPs, and AgNPs-20.

		1%-$AgSiO_2$NPs	10%-$AgSiO_2$NPs	AgNPs-20
**Elemental Composition**	**Trace Metal Analysis**‡ (purity %)	99.98 ± 0.05	99.98 ± 0.25	99.01 ± 0.05
	**Carbon Content*** (w/w %)	0.048 ± 0.200	0.048 ± 0.177	0.197 ± 0.491
	**Stoichiometry by XPS**	$SiO_{2.02}$	$SiO_{2.07}$	Ag3d
**Elemental Composition**	**Recombinant Factor C** (EU/mg)	2.647	23.93	1.789
	**Sterility**† (bacteria/mg)	0	0	0

XPS, X-ray photoelectron spectroscopy; ICP-MS, inductively-coupled plasma mass spectrometry; *elemental plus organic carbon content; † Pharmacopeia Protocol for Sterility WHO Document QAS/11.413 FINAL; ‡ weight percentage of total metals detected. These data have been previously published by Beltran-Huarac and coauthors (Beltran-Huarac et al., 2018).

Supplementary Table 3: Dynamic light scattering (DLS) measurements of 1%-$AgSiO_2$NPs, 10%-$AgSiO_2$NPs, and AgNPs-20 suspensions in cell culture medium or balanced salt solution (BSS).

Dispersion medium: EpiLife + 1% EDSG					
**NPs**	**Sonication time (min)***	**Time after sonication (h)**	**Hydrodynamic diameter (nm)**	**Polydispersity Index (PDI)**	**P-value**
**Au**	-	1	227.2 ± 11.2	0.202 ± 0.061	0.729
		24	229.5 ± 11.2	0.243 ± 0.044	
**AgNPs-20**	3.4	1	354.5 ± 89.8	0.586 ± 0.081	0.822
		24	347.2 ± 80.0	0.593 ± 0.053	
**1%-$AgSiO_2$NPs**	3.2	1	196.2 ± 4.1	0.251 ± 0.021	0.210
		24	177.0 ± 20.6	0.271 ± 0.029	
**10%-$AgSiO_2$ NPs**	4.7	1	668.3 ± 242.2	0.523 ± 0.081	0.012*
		24	294.3 ± 38.3	0.291 ± 0.033	
Dispersion medium: BSS					
**NPs**	**Sonication time (min)***	**Time after sonication (h)**	**Hydrodynamic diameter (nm)**	**Polydispersity Index (PDI)**	**P-value**
**Au**	-	1	153.5 ± 19.0	0.270 ± 0.051	0.164
		24	130.6 ± 5.5	0.320 ± 0.034	
**AgNPs-20**	3.4	1	134.2 ± 52.6	0.228 ± 0.059	0.724
		24	145.5 ± 55.2	0.279 ± 0.015	
**1%-$AgSiO_2$NPs**	3.2	1	511.9 ± 40.0	0.283 ± 0.128	0.317
		24	530.9 ± 13.7	0.347 ± 0.131	
**10%-$AgSiO_2$ NPs**	4.7	1	253.6 ± 25.31	0.682 ± 0.133	0.533
		24	217.9 ± 51.74	0.587 ± 0.075	
Dispersion medium: DMEM					
**NPs**	**Sonication time (min)***	**Time after sonication (h)**	**Hydrodynamic diameter (nm)**	**Polydispersity Index (PDI)**	**P-value**
**Au**	-	1	43.2 ± 11.5	0.455 ± 0.005	0.864
		24	44.5 ± 1.8	0.500 ± 0.024	
**AgNPs-20**	3.4	1	283.1 ± 102.1	0.533 ± 0.091	0.157
		24	206.7 ± 63.8	0.541 ± 0.114	
**1%-$AgSiO_2$NPs**	3.2	1	156.7 ± 43.4	0.417 ± 0.178	0.988
		24	157.0 ± 14.8	0.649 ± 0.112	
**10%-$AgSiO_2$ NPs**	4.7	1	804.1 ± 92.6	0.363 ± 0.079	0.431
		24	862.3 ± 145.5	0.227 ± 0.054	

* Sonication time is indicated for a 1 mL volume of the NP suspension; Unpaired t test with Welch's correction was performed to compare 1- and 24-hours post-suspension for each nanoparticle. *$P < .05$, considered statistically significant.

Supplementary Figure 1: **Cell viability measured by Calcein-AM assay was dose dependently reduced following treatment with three silver nanoparticles.** The Calcein-AM assay revealed similar cell viability results for the hTCEpi cells (A-C) and RCFs (D-F). The AuNPs (5 µg/mL), DW and saponin (1 mg/ml) were used as negative, vehicle and positive controls, respectively. The data represent mean ± standard deviation (SD), n ≥ 3. Error bars represent the SD and asterisks indicate significant differences compared with the vehicle controls. *P < .05, repeated measures one-way ANOVA followed by Tukey's multiple comparisons.

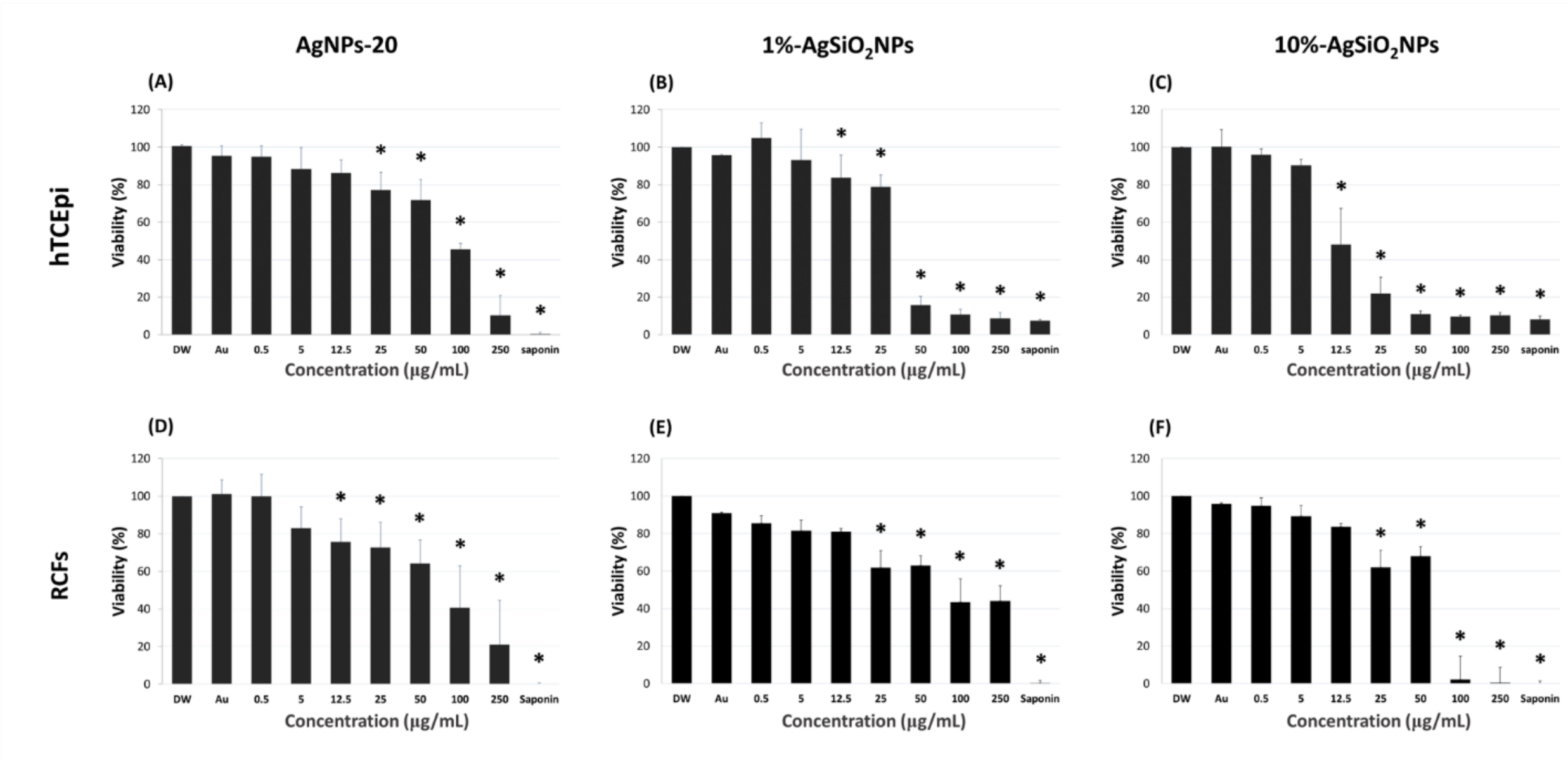

Supplementary Figure 2: **No significant differences in clinical scoring were identified between groups at all time points.** External photos (A) and clinical scoring (B) showed conjunctival hyperemia, chemosis, corneal opacity and iris hyperemia transiently until the corneal epithelial wounds healed. Repeated measures two-way ANOVA followed by Tukey's multiple comparisons was performed to determine statistical differences of the clinical scorings between groups and time points.

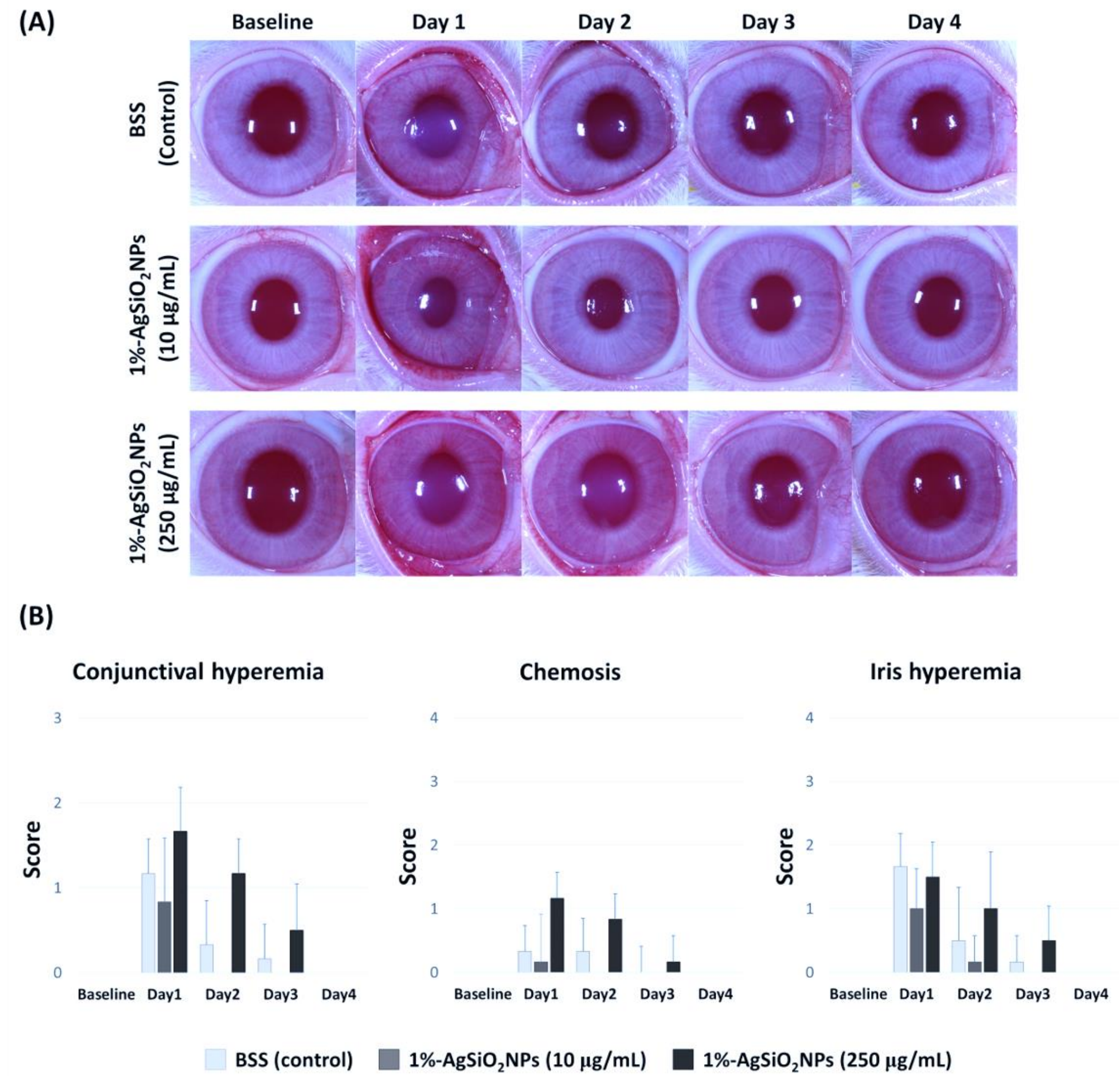

Supplementary figure 3: The 1%-$AgSiO_2$NPs were observed in the iris stroma and anterior chamber. (A) Autometallography showed stained 1%-$AgSiO_2$NPs (black arrows) in the iris stroma of unwounded eye. The 1% toluidine blue was applied for the counter stain. (B) Dark field hyperspectral microscopy showed 1%-$AgSiO_2$NPs (white arrows) in the anterior chamber of wounded eye as well as in the cornea. S, stroma; En, endothelium; AC, anterior chamber.

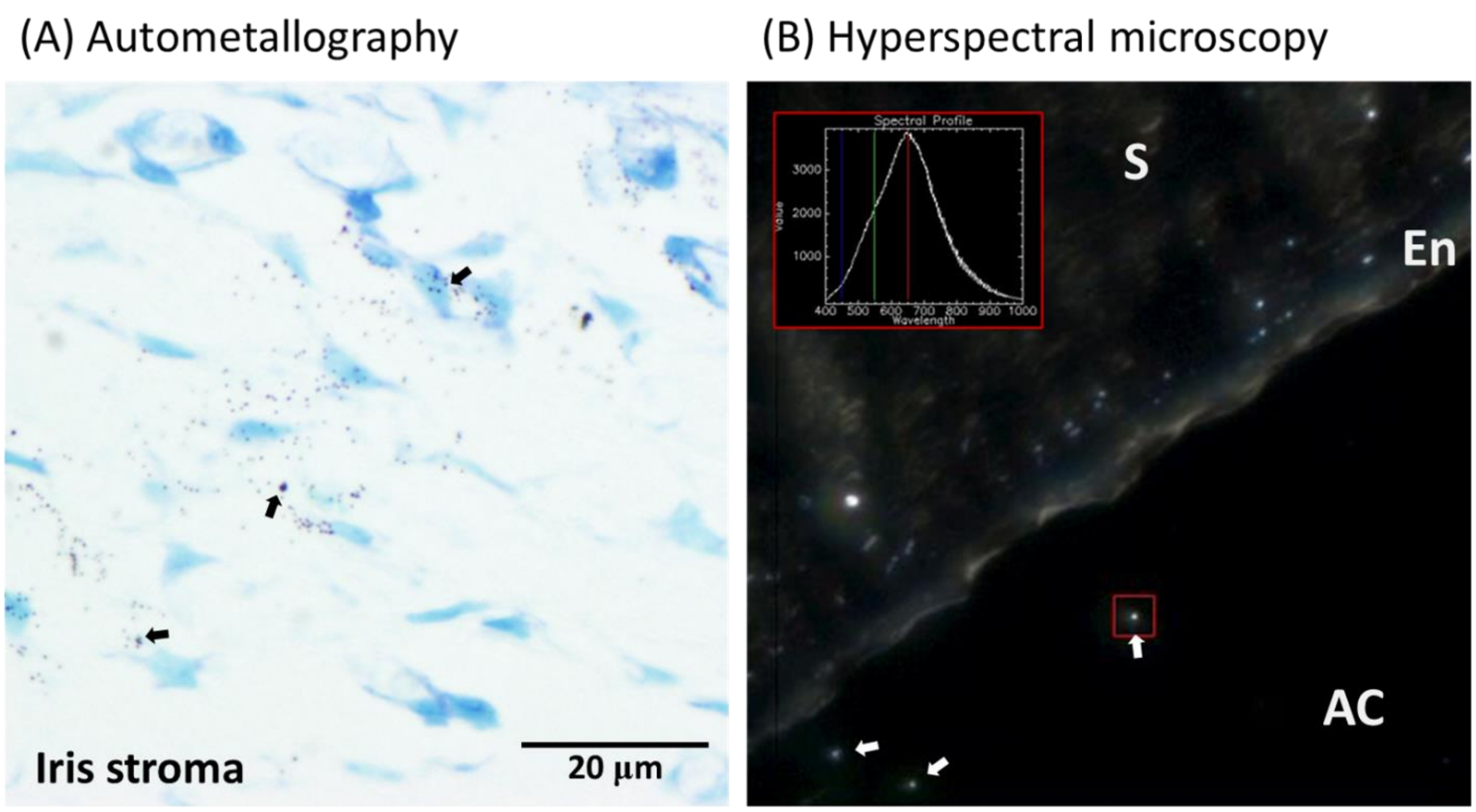

Supplement: Supplementary file 1 [file NIHMS2203212-supplement-Supplementary_data.pdf]
